# Recovery of Immunological Homeostasis Positively Correlates Both with Early Stages of Right-Colorectal Cancer and Laparoscopic Surgery

**DOI:** 10.1371/journal.pone.0074455

**Published:** 2013-09-09

**Authors:** Mario Ferri, Simone Rossi Del Monte, Gerardo Salerno, Tommaso Bocchetti, Stefano Angeletti, Florence Malisan, Patrizia Cardelli, Vincenzo Ziparo, Maria Rosaria Torrisi, Vincenzo Visco

**Affiliations:** 1 Surgical and Medical Department of the Clinical Sciences, Biomedical Technologies and Translational Medicine, Faculty of Medicine and Psychology University of Rome “Sapienza”, Rome, Italy; 2 Department of Clinical and Molecular Medicine, Istituto Pasteur-Fondazione Cenci Bolognetti, Faculty of Medicine and Psychology University of Rome “Sapienza”, Rome, Italy; 3 Sant’Andrea Hospital, Rome, Italy; 4 Laboratory of Immunology and Signal Transduction, University of Rome ‘Tor Vergata’, Rome, Italy; University of Oslo, Norway

## Abstract

Differences in postoperative outcome and recovery between patients subjected to laparoscopic-assisted versus open surgery for colorectal cancer (CRC) resection have been widely documented, though not specifically for right-sided tumors. We investigated the immunological responses to the different surgical approaches, by comparing postoperative data simultaneously obtained at systemic, local and cellular levels. A total of 25 right-sided CRC patients and controls were managed, assessing -in the immediate followup- the conventional perioperative parameters and a large panel of cytokines on plasma, peritoneal fluids and lipopolysaccharide (LPS)-stimulated peripheral blood mononuclear cells (PBMC) tissue cultures. A general better recovery for patients operated with laparoscopy compared to conventional procedure, as indicated by the analysis of typical pre- and post-surgical parameters, was observed. The synchronous evaluation of 12 cytokines showed that preoperative plasma levels of the proinflammatory cytokines IL-6, IL-8, IL-1β, TNFα were significantly lower in healthy donors versus CRC patients and that such differences progressively increase with tumor stage. After surgery, the IL-6 and IL-8 increases were significantly higher in open compared to laparoscopic approach only in CRC at early stages. The postsurgical whole panel of cytokine levels were significantly higher in peritoneal fluids compared to corresponding plasma, but with no significant differences depending on kind of surgery or stage of disease. Then we observed that, pre- compared to the corresponding post-surgery derived LPS-stimulated PBMC cultures, produced higher supernatant levels of the whole cytokine panel. In particular IL-6 *in vitro* production was significantly higher in PBMC derived from patients subjected to laparoscopic versus open intervention, but -again- only in CRC at early stages of disease. Our results thus show that laparoscopy compared to open right resection is associated with a shorter compromission of the immunological homeostasis, mainly in early stages of right-CRC patients.

## Introduction

Colorectal cancer (CRC) is the third most common cause of cancer-related mortality worldwide, whose standard therapy remains surgery [1], [2]. The incidence of right- compared to left-sided colon cancers have increased during the last three decades, so that at present right-sided tumors represent approximately 50% of all colon cancer [3], [4]. In addition, some authors detected a modestly better prognosis in left-sided compared to right-sided tumors, although this argument remains matter of debate [4]–[6].

Laparoscopic segmental colectomy is a well established surgical technique that, compared to traditional open colectomy, is characterized by less morbidity, less postoperative pain, a fast postoperative recovery and a short hospital stay, even though both methods are associated with the same radical surgery [7], [8]. However, although the short-term benefits of laparoscopy compared to conventional open surgery are now well recognized in the management of different colorectal diseases, this remains a controversial issue for the right-sided tumors [9], [10].

It is now generally accepted that the extent of surgical trauma influences the acute inflammatory response [11], which in turn may be responsible not only for possible complications (such as infections and long-term abdominal adhesions) [12], but also for tumor metastasis formation [9], [13], [14]. Although only few and disputable data on the postoperative recovery of immune homeostasis in CRC patients have been published, it is now well accepted that an earlier restoring of immune competence can influence disease recurrence and prognosis [9], [13], [15], [16]. Moreover, in the recent past, there are convincing signs that immunological response to cancer and alterations in the cellular immune system could be significantly affected by surgery [17].

It is now well recognized that -beyond the C-Reactive Protein (CRP), which is probably the main serum marker for inflammation- cell count for white blood cells (WBC) and polymorphonuclear cells (PMN) represents a good support for postoperative immunological evaluation of cancer patients [18], [19]. However, even more than variations in cell numbers, it seems very important to verify possible alterations in their functions, such as the secretive activity [20]. In particular, a more exhaustive analysis of the inflammatory processes following the injury may currently be provided by the assessment of cytokines.

Therefore, with the aim to investigate the immunological responses to different surgical approaches in patients undergoing right colon resection for cancer, we performed -for the first time- a comparison between short-term data on the cytokine release simultaneously obtained at systemic, local and cellular levels. In order to arrange a comprehensive evaluation of the postoperative profiles displayed in those patients, we investigated the cytokine concentration both in plasma and in peritoneal fluids collected at different times after the conventional postoperative placement of a drainage system, because the local peritoneal response can be considered an important determinant of postsurgical recovery [21]. Nevertheless, as previously reported [22], a further convincing index of the inflammatory state next to the surgery could be represented by the analysis of the peripheral blood mononuclear cells (PBMC) capacity to secrete cytokines *in vitro*, in presence of a lipopolysaccharide (LPS)-mediated stimulation.

A new method using multiplex biochip array for simultaneous detection of a large panel of multiple cytokines in each single sample has recently attracted a considerable interest [23]. Herein the synchronous assessment of 12 different cytokines was performed, including inflammatory proteins such as tumor necrosis factor–α (TNF-α), IL-1 (α and β), IL-2, IL-4, IL-6, IL-10 and interferon-γ (IFN-γ), chemokines (IL-8, and monocyte chemoattractant protein–1: MCP-1) and growth factors (vascular endothelial growth factor: VEGF and epidermal growth factor: EGF). All data were finally evaluated in comparison with the clinicobiological features and the conventional perioperative parameters collected in the immediate followup.

Collectively, our results thus show that laparoscopy compared to open right resection is associated with a shorter compromission of the immunological homeostasis, but mainly in early stages of right-CRC patients.

## Materials and Methods

### Study Design

The study design was approved by the university ethical committee and all the enrolled patients gave informed consent (Prot. C.E. 843/2012). Participants provide their written informed consent to participate in this study.

Between May 2008 and April 2011, all patients diagnosed with right colon cancer at the Surgery Unit of Sant’Andrea Hospital were assessed and 25 of them were consecutively enrolled for this study, based on the following criteria.

The eligibility criteria provided patients affected by primary colonic adenocarcinoma, scheduled for elective resection by right colectomy, enrolled prior any neoadjuvant therapy and subjected to postsurgical placement of a low-vacuum drainage system for peritoneal fluids collection.

Exclusion criteria were: a pre-existing history of abdominal surgery or cancer and inflammatory diseases, the outset of intra- and post-surgical complications, the admnistration of intra- and postoperative blood transfusions interfering with the immune response, pre- or intra-operative detection of peritoneal carcinomatosis, emergency surgery or abdominal complications. The study group consisted of 25 patients (12 men and 13 women aged from 59 to 83 years) that were managed after surgery (10 subjected to minimally invasive and 15 to conventional open procedures) following the same protocol: (a) serial blood samples were collected preoperatively, at 6, 24, 48 and 72 hrs (hours) postoperatively; (b) postsurgical peritoneal fluids collection was performed at 24 and 48 hrs; (c) mononuclear cells were derived from peripheral blood (PBMC) before and 24 hrs after surgery and cultured for additional 48 hours: then supernatants were collected for each culture. We achieved two different control groups. The first included 10 healthy volunteers (5 men and 5 women aged from 47 to 70 years), whose blood samples were withdrawn for simultaneous and serial dosages of cytokines and for PBMC cultures (as above). An additional control group consisted of 2 supplemental patients who underwent right colectomy by open surgery for non-neoplastic colorectal diseases and were then processed applying exactly the same procedures used for cancer patients, including plasma, peritoneal fluids collection and PBMC cultures.

The following parameters were recorded in all patients: age, gender, body mass index (BMI), American Society of Anesthesiologists (ASA) score, length of incision, estimated blood loss, operating time, time to resumption oral intake, amounts of analgesics used for pain control, time to first bowel movement, time to deambulation, hospital stay. The clinicopathological characteristics of the patients were obtained from the pathologic reports after surgery. Tumors were classified as proposed by the 6^th^ edition of the TNM classification of malignant tumors from the International Union Against Cancer (UICC). Patients were then stratified in two groups according to tumor stage: 13 “early” patients for I-II stages of disease and 12 “advanced” patients for III-IV stages of disease.

### Surgery

All patients underwent right colectomy and were assigned to two possible surgical approaches: (a) a minimally invasive laparoscopic technique or (b) a conventional open surgery. All resections were performed according to accepted oncologic principles with the same extent of resection for both groups, including high vessel ligation, nodal clearance and adequate bowel margin. For laparoscopic resections, pneumoperitoneum was created and intracorporeal approach through 4 abdominal ports (1–1,2 cm of diameter) was used for surgical manoeuvres. The bowel was exteriorized through a small periumbilical incision (5–7 cm length) for resection and anastomosis. The open colectomy was done via a midline incision (16–20 cm length) using conventional surgical techniques. In all patients restoration of bowel continuity was accomplished with a stapled ileo-transverse anastomosis and a low-vacuum drainage system was placed for peritoneal fluids collection.

All patients received i.v. morphine for 24 hrs postoperatively and i.v. Non Steroidal Anti-Inflammatory Drugs (NSAIDs) on request. Patients were allowed to take liquid when their bowel sounds become audible, and they were progressed to a solid diet as tolerated. Mobilization and catheter removal was encouraged as soon as the patient was physically capable. Patients were discharged when fully ambulatory and tolerant of a solid diet.

### Blood, Peritoneal and Cells Samples

Four milliliters of heparinized venous blood samples were collected, including patients and controls, prior and 6, 24, 48 and 72 hrs after surgery (T0–6–24–48–72). After centrifugation, samples were stored at −80°C until assays to determine the cytokines concentration, C-reactive protein (CRP, acute phase marker) the counts of total leukocytes, lymphocytes and polymorphonuclear granulocytes.

24 and 48 hrs after surgery, peritoneal fluids were collected and then cytokine concentrations were measured as described below. Around ten milliliters of 24 hrs blood samples were also collected to isolate peripheral blood mononuclear cells (PBMC) using density gradient centrifugation over Ficoll-Paque™ PLUS (Amersham Biosciences/GE Healthcare), either from 25 CRC patients or from 10 healthy donors used as controls. Cells were then cultured in triplicate at the density of 1×10^6^ cells/ml on 24 wells/plate (Becton Dickinson, Oxnard, CA) in RPMI-1640 supplemented with 10% fetal bovine serum (FCS) and antibiotics and stimulated with 1.0 µg/ml LipopolysaccharideS (LPS) (all reagents from Sigma Chemicals Co., St.Louis, MD). After additional 48 hrs of culture, cell supernatants were obtained and collected to determine cytokines concentration.

The perioperative inflammatory profiles of the all patients were then investigated, at different times evaluating 12 cytokine levels in plasma, peritoneal fluids and cell culture supernatants collected as described above.

### Determination of Cytokine Levels

For simultaneous assessment of the following 12 cytokines: IL-1α, IL-1β, IL-2, IL-4, IL-6, IL-8, and IL-10; vascular endothelial growth factor (VEGF); epidermal growth factor (EGF); tumor necrosis factor–α (TNF-α); interferon-γ (IFN-γ) and monocyte chemoattractant protein–1 (MCP-1), a multiplex biochip array in Evidence equipment (Randox Labs. Ltd. Crumlin, UK) was used as previously reported [23]. Assays were performed on plasma, peritoneal fluids and PBMC culture supernatants, following the manufacturer’s instructions. The analyte’s concentration present in the sample was calculated automatically using routinely generated calibration curves (Evidence Software version 1.4).

### Statistical Analysis

The results are expressed as mean ± standard deviation (SD), mean ± standard error (SEM) or median and interquartile range.

Statistical significance was defined at *p*<0,05.

The normality of data distribution was checked with Shapiro-Wilk test, and non-normally distributed data were logarithmically transformed before analysis. Comparison between and within groups were done by Mann-Whitney or Unpaired tests and Paired or Wilcoxon-Pratt tests respectively. The Pearson or Spearman correlation coefficient was used to assess relations between the variables. For categorical variables Chi-square or Fisher test was used. Statistical analysis was performed using the Graph Pad Prism 5.0 (GraphPad Software Inc., San Diego, CA, USA), and SigmaPlot V.1 1 (Copyright © 2008 Systat Software, Inc.).

## Results

### Pre- and Post-surgical Patients Characteristics

25 right-sided CRC patients -uniformly stratified in 13 early (I-II) and 12 advanced stage (III-IV) of disease- were enrolled for laparoscopic or conventional open intervention, which defined two different groups comparable with respect to numerosity, age, gender, ASA score and BMI (table 1).

**Table 1:** Demographic and clinico-pathological characteristics of patients.

Parameter	Laparoscopy (n = 10)	Conventional (n = 15)	P value (Student’s T test)
Age (yr)	70±8	73±15	ns
Sex: M(F)	5(5)	6(9)	ns
ASA	2.4±0.5	2.4±0.6	ns
BMI Kg/mq^2^	22±2.23	23.5±2.89	ns
TNM			
stage I	0	0	ns
stage II	5	8	ns
stage III	3	6	ns
stage IV	2	1	ns

Data are number of patients or mean ± standard deviation.

ASA: American Society of Anesthesiologist.

BMI: Body Mass Index.

ns: not significant.

In order to compare the perioperative parameters of laparoscopy versus conventional open procedures, length of incision, duration of intervention and blood loss as well as starting of first bowel movement and solid diet, analgesic requirement, time to deambulation and hospital stay were collected (table 2). Altogether, they show a general better recovery for patients operated with laparoscopy, as indicated by a significant difference between the two surgical approaches in length of incision (*p*<0.0001), (*p* = 0.0008), time of solid diet (*p* = 0.005), time to deambulation (*p* = 0.003) and hospital stay (*p* = 0.0002), even though the operative time was longer (*p* = 0.0008).

**Table 2:** Surgical details and postoperative recovery parameters.

Parameter	Laparoscopy (n = 10)	Conventional (n = 15)	P value (Student’s T test)
Surgery*			
Length of incision (cm)	6.8±0.8	15.9±2.3	<0.0001
Operative time (min)	178.0±41.4	123.3±29.6	0.0008
Blood loss (ml)	50.0±33.3	39.3±44.6	ns
Post Surgery**			
Time to first bowel movement	4.0(3.0–4.5)	3.0(2.5–4.5)	ns
Time to solid diet	4.0(3.75–5.0)	3.0(2.5–3.5)	0.005
Analgesic requirement	1.5(0.75–0.25)	2(1.0–3.0)	ns
Time to deambulation	2.0(1.0–2.0)	3.0(2.0–3.0)	0.003
Hospital stay	6.0(5.0–7.0)	9.0(8.0–10.0)	0.0002

* Data are mean ± standard deviation.

** Data are median of postoperative days (range IQR).

ns: not significant.

Pre- and postoperative numbers of WBC and PMN were evaluated in the different groups and controls. In the first 72 hrs after surgery compared to preoperative analysis as well as to healthy donors, a significant increase in cell number was observed (peak between 6 and 24 hrs), which tended to normalize, independently of either kind of surgery or stage of disease. Similarly, the plasmatic levels of CRP show progressive increases, starting from 6 hrs and peaking 48 hrs after intervention. However, this analysis shows significant differences with respect to the different surgical approaches, but not to based-tumor stage subgroups. In particular, CRP levels after 24 hrs were significantly higher (*p*<0.01) in patients subjected to open versus laparoscopic surgery, whereas those differences -even though always detectable- resulted not statistically significant in the other times (6, 48 and 72 hrs).

### Presurgical Proinflammatory Cytokine Levels Directly Correlate to the Stage of Disease

In order to better compare the two different surgical approaches, we simultaneously measured the plasmatic levels of 12 cytokines from blood derived from CRC patients before and after resection.

**Figure 1:**
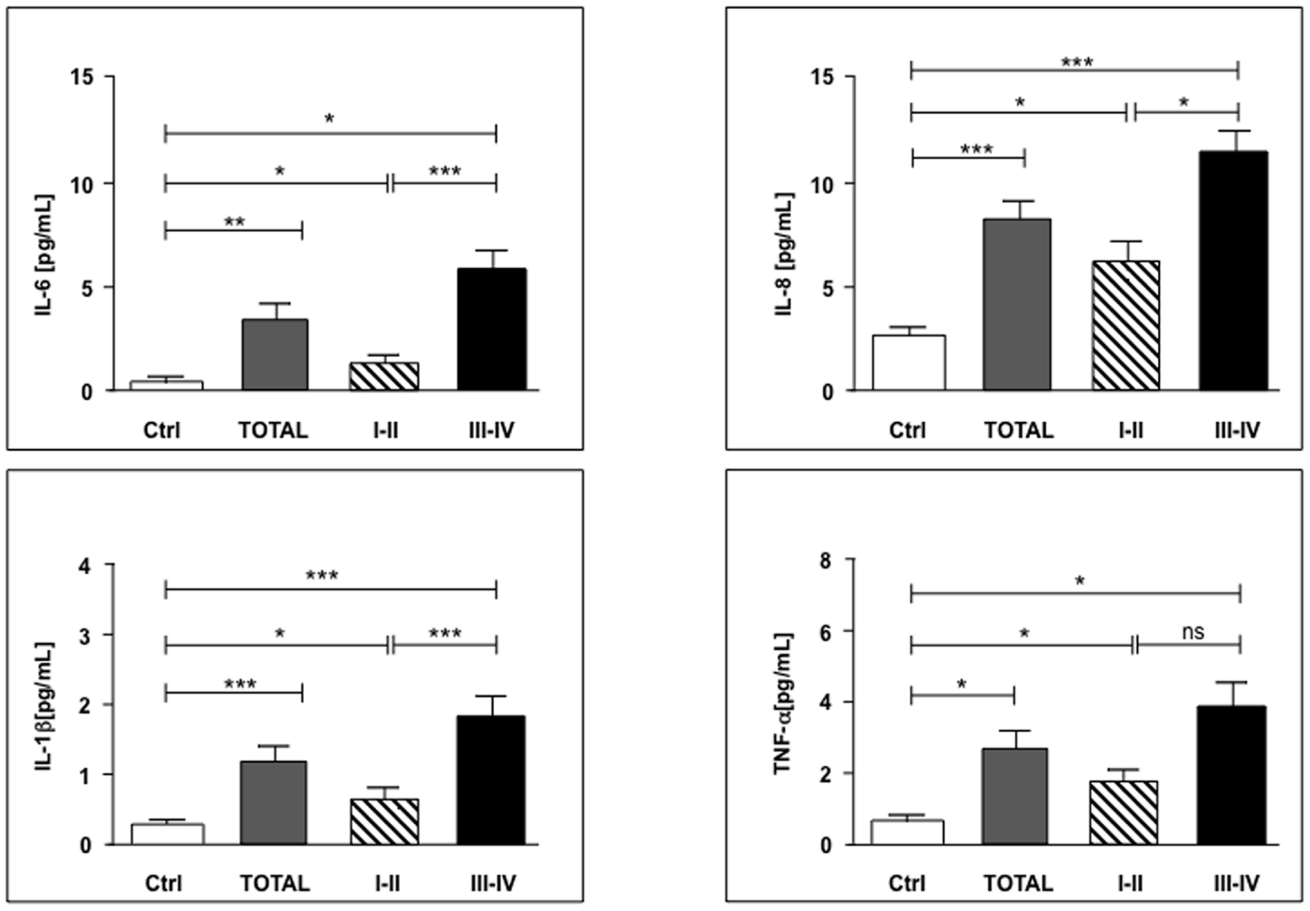
Presurgical proinflammatory IL-6, IL-8, IL-1β and TNFα plasma levels significantly correlate to the stages of CRC. Cytokines levels were measured from plasma of control group (Ctrl) or 25 CRC patients before surgery (Total), including 13 early (I-II) and 12 advanced stage (III-IV) of disease. Values are expressed as mean ± SEM and statistics were performed using unpaired Student’s T test. P values are assumed as statistically significant at *p*<0.05 (*); *p*<0.01 (**); *p*<0.001 (***). ns = not significant.

Presurgical (T0) levels of IL-6, IL-8, IL-1β, TNFα (baselines) were significantly (*p*<0.05) lower in healthy donors versus CRC patients and these differences progressively increase with tumor stage (Fig. 1), whereas in the other cytokines no significant differences were observed (data not shown). In particular, except for TNFα, the all cited cytokines show a significant increase in the baseline levels when we compared early (I-II) to advanced (III-IV) stages of disease (*p*<0.05) (Fig. 1).

Interestingly, additional control patients -carrying non neoplastic gastrointestinal pathologies- show cytokine levels similar to healthy volunteers (data not shown). Moreover, the baselines cytokine levels were comparable for both laparoscopic and open resection groups (data not shown).

### IL-6 and IL-8 Postsurgical Plasma Levels are Lower after Laparoscopy Compared to Open Surgery Only in Early Stages Patients

**Figure 2:**
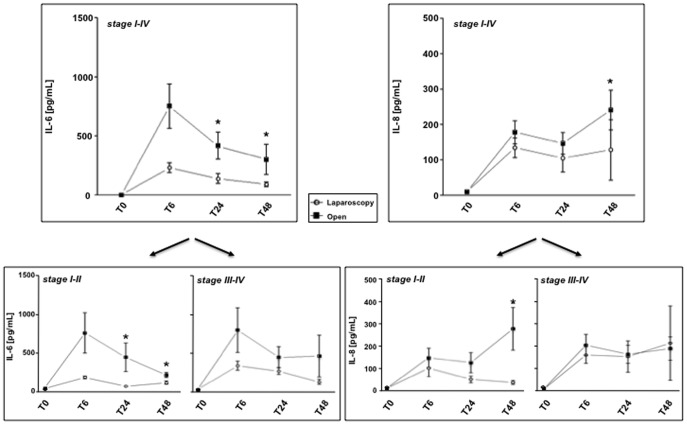
Postsurgical IL-6 and IL-8 plasma level increases are significantly lower after laparoscopy compared to open surgery, at early stages of CRC. Pre- (T0) and postoperative (T6–48) IL-6 and IL-8 levels levels were measured from plasma of 25 CRC patients before surgery (stage I-IV), including 13 early (stage I-II) and 12 advanced (stage III-IV) stage of disease. Values are expressed as mean ± SEM and statistics were performed using unpaired Student’s T test. P values are assumed as statistically significant at *p*<0.05 (*).

When we compared postoperative plasma levels of the whole cytokine panel, a significant difference in laparoscopic versus open resection patients was observed only for IL-6 and IL-8 (Fig. 2). Surprisingly, even though our data displayed distinct kinetics for each cytokine, we observed that increases in open versus conservative approaches for both IL-6 and IL-8 can be clearly observed only in CRC early stages, whereas -in advanced tumors- the choice of surgery did not further influence the postoperative cytokine levels. In particular, postsurgical IL-6 detection displayed significant increases after 24 and 48 hours in patients subjected to conventional invasive technique compared to laparoscopy, although the peak of cytokine production was achieved at 6 hours in both groups (Fig. 2). The significant increments of IL-6 at T24 e T48 mainly attended in the early stages, whereas in advanced stage of disease no substantial differences between laparoscopic and open surgery groups were observed. In addition, similarly to IL-6, even IL-8 postsurgical levels were generally higher in open compared to laparoscopic surgery, but this difference resulted significant at 48 hours only and, again, especially for the early stages of disease (Fig. 2). Notably, a progressive decrease in IL-8 was detected until 48 hours following laparoscopy but not after invasive intervention (Fig. 2).

For the other two cytokines that were significantly different in the baseline preoperative detection, (IL-1β and TNFα), no significant changes from conservative versus conventional surgical technique have been detected (data not shown). The levels of the other eight cytokines, whose presurgical values in CRC and non neoplastic patients were not significantly different compared to healthy donors, increased after tumor resection, but independently of the kind of surgery or stage of disease (data not shown). Interestingly, IL-6 increases directly correlated with CRP levels, either 24 hrs (r^2^ = 0.51 with *p* = 0.0086) or 48 hrs (r^2^ = 0.49 with *p* = 0.012) after surgery, whereas for the remaining plasmatic cytokines no relationships with CRP were observed (data not shown).

Cytokine levels of CRC patients in peritoneal fluids collected 24 and 48 hours after surgery were also measured. A significant increase in cytokine concentration compared to corresponding plasma, ranging from 400-fold increase for IL-6 to 2-fold increase for MCP-1 (data not shown) was remarked. However no significant differences in the whole panel of cytokines -depending on time (T24 and T48) and kind of surgery or stage of disease- was ever detected (data not shown).

### IL-6 *in vitro* Production by PBMC, upon LPS Stimulation, is Significantly Increased after Laparoscopy, only in Early Stages of Disease

**Figure 3:**
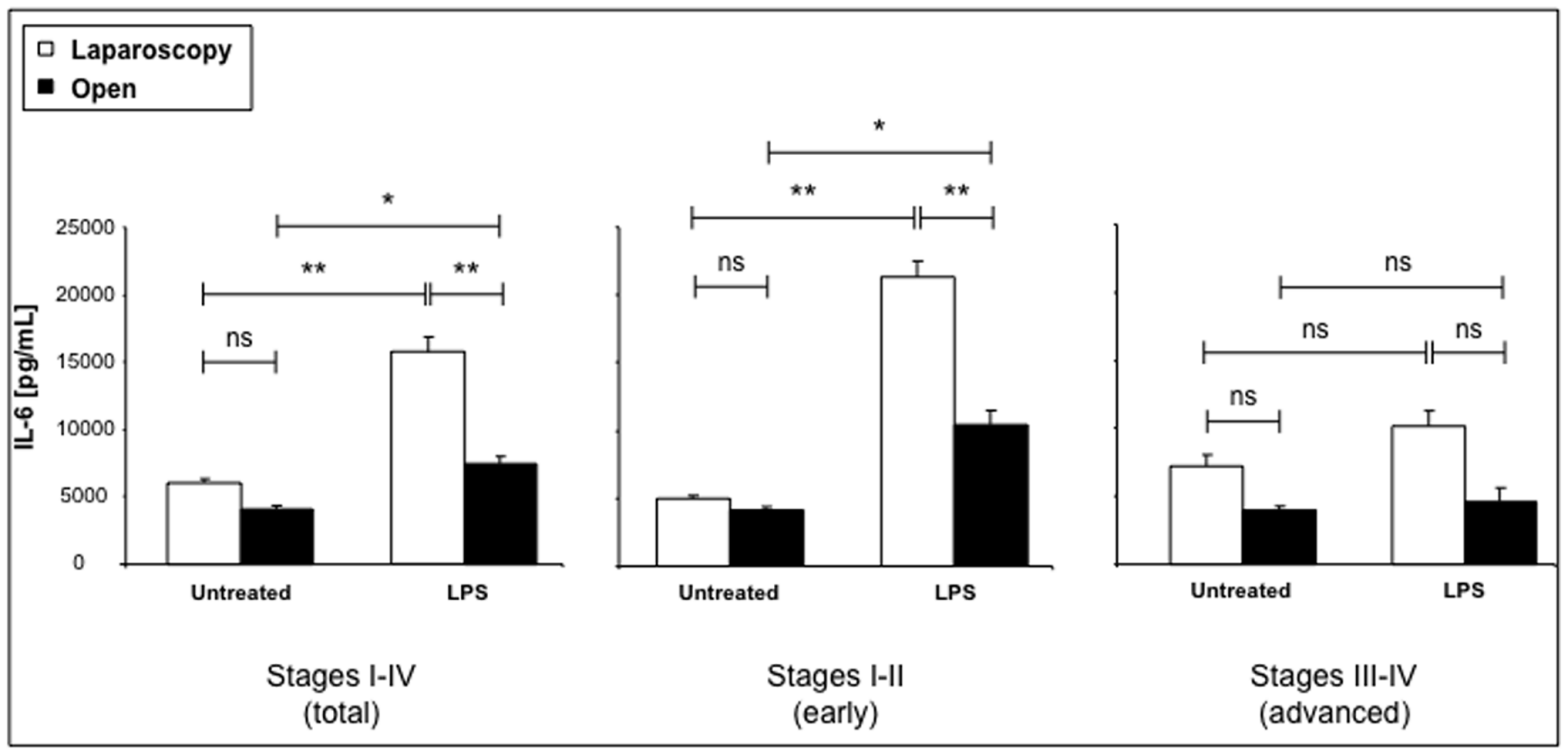
Postsurgical IL-6 *in vitro* production by PBMC is significantly stimulated by LPS, at early stages CRC. IL-6 levels were measured in supernatants collected from LPS-stimulated PMBC cultures. Samples derived from 25 CRC patients (total, stage I-IV), including 13 early (stage I-II) and 12 advanced (stage III-IV) stage of disease. Values are expressed as mean ± SEM and statistics were performed using unpaired Student’s T test. P values are assumed as statistically significant at *p*<0.05 (*); *p*<0.01 (**); *p*<0.001 (***). ns = not significant.

The capacity of PBMC derived from healthy donors, laparoscopic or open surgery patients to produce cytokines was further analyzed in vitro, measuring the cytokines in the supernatant of LPS-stimulated PBMC both before and 24 hrs after surgery. First, we observed higher cytokine levels in supernatants collected from LPS-stimulated PBMC compared to unstimulated cell cultures derived from blood samples, either in healthy donors or in CRC patients before and 24 hrs after surgery (data not shown). Then, presurgery compared to the corresponding postsurgery-derived cell cultures produced higher supernatant levels of the whole cytokine panel (data not shown). Within the presurgical samples, cytokine production was higher in supernatants derived from early stages than in those derived from advanced stage of CRC (data not shown). On the other hand, postsurgical PBMC, derived from CRC patients subjected to laparoscopic versus open resection, produce higher amounts of cytokines and increased efficiency of LPS stimulation (ranging from 15-fold increase for IL-6, to 2,5-fold increase for MCP-1 in LPS-stimulated vs unstimulated samples). In particular, the IL-6 detection in supernatants of cultured PBMC derived from CRC patients 24 hours after surgery is showed in Figure 3, being the more representative of the whole panel of cytokines. LPS induced a significant enhancement in cytokine production either after laparoscopic or open intervention, even though the efficiency of stimulation was more significant in supernatants of PBMC cells derived from patients subjected to laparascopic vs open surgery (*p*<0.01 vs *p*<0.05) (Fig. 3).

It is noteworthy that, when we compared LPS-mediated effects in postsurgical cultures derived from early versus advanced stage of disease, a significant stimulation in IL-6 secretion has been found exclusively in the early (I-II), whereas no substantial differences were observed in the advanced stages of disease, whose values (±LPS) were always lower compared to the corresponding early stages (Fig. 3). In addition, the LPS stimulating effect was more significant in cells derived from laparoscopic (*p*<0.01) compared to open (*p*<0.05) procedure, in CRC patients at early stages of disease (I-II), whereas such differences were not significant when laparoscopy was compared to open surgery group in patients affected by CRC at advanced stages (III-IV) (Fig. 3). When we compared untreated control and LPS stimulated supernatants, no significant differences were observed in controls from open and laparoscopic surgery, whereas a significant increase in LPS stimulated cells derived from patients subjected to laparoscopic versus open intervention was detected (*p*<0.01) (Fig. 3).

Similarly to IL-6, also for IL-8, IL-1β, TNFα and MCP-1, LPS-induced significant increases (*p*<0.05) in supernatants of cultured cells have been found, but independently of the kind of surgery and the stage of CRC patients (data not shown). In the other remaining cytokines detected in supernatants from cultured PBMC of cancer patients, LPS stimulation did not produce any significant increase of production and, in addition, no substantial differences have been observed comparing subjects undergone to conservative or open surgery and bearing early or advanced stage of disease (data not shown).

## Discussion

In this study, we show for the first time the simultaneous analysis of 12 cytokines from systemic (blood), local (peritoneal fluids) and cellular (supernatants from cell cultures) samples derived from right colon cancer patients subjected to different surgical approaches for tumor resection. 25 patients affected by cancer of the right colon, 13 bearing early stage and 12 advanced stage of disease, destined to minimally invasive laparoscopy or conventional open intervention, were investigated in the short term pre- and postoperative period for cytokine production. In fact, although a causative connection between colon cancer and inflammation has been well established [24], the possible implications of cytokine short-term responses, in patients subjected to different surgical procedures for right colorectal cancer resection are not yet clarified. Our results show that the recovery of immune homeostasis of CRC patients subjected to colectomy could not only be related to the kind of surgery [25], but also to the stage of tumor disease.

The analysis of conventional acute phase markers shows -as expected [2], [20], [25], [26]- a significant postoperative enhancement in WBC and PMN number, within 72 hours after surgery, which tended progressively to normalize, but independently of either kind of surgery or stage of disease. On the contrary, the CRP increments, starting from 6 hrs and peaking 48 hrs after intervention and being more elevated in open versus laparoscopic surgery, appeared significantly different with respect to the kind of intervention (as expected cfr. [2], [27]–[29]), but not to based-tumor stage subgroups. Even though those markers are considered a convincing index of the acute inflammatory response to surgical trauma, further investigations are required to establish the role of different surgical approaches in determining -not only the postoperative recovery of immune functioning- but more generally the outcome in CRC patients [24]. At the light of those observations, the postsurgical short-term evaluation of systemic, local and cellular cytokines profile may become an independent variable in the prognostic assessment of right colon cancer affected patients. In fact, as previously reported [19], [30], [31], some cytokines could be considered not only a biomarker of the acute-phase response, tightly related to the degree of surgical trauma, but are also potentially able to influence the short-term postoperative outcome [32], the restoration of immune homeostasis [13], the incidence of infectious complications and local recurrences [14], [33] and the long-term oncologic results [16].

Our presurgical analysis shows that some cytokine levels (IL-6, IL-8, IL-1β and TNFα) were significantly higher in plasma of patients affected by colorectal cancer compared to healthy donors and these differences progressively increase in advanced tumor stages. This is not surprising, because several investigations revealed a direct correlation between increasing plasmatic levels of proinflammatory cytokines and colorectal tumor stage and progression, particularly concerning IL-6, whose expression is frequently increased in cancer disease and considered a predictor of poor outcome [23], [24], [34]–[41]. Our data confirmed previous investigations indicating a relationship between IL-6 secretion (peaking at 6 hours after surgery) and the following CRP production (peaking at 48 hours after surgery) [27], [42]. Although rising opinions support the role of CRP as a predictor of lower survival rates in patients with several cancers, including CRC [43], no definitive results have been published regarding its effective value as an independent variable on postsurgical recovery [29]. Nevertheless, earlier reports showed some conflicting findings on the plasmatic cytokine presurgical levels, probably imputable to differences in the performed methodologies [23].

It has been proposed that postsurgical plasma cytokine levels may be considered an accurate marker of the overall acute-phase response and used to monitor the impact of surgery, which is potentially different according to the distinct operative procedures [20], [44], [45]. It seems to be in agreement with our results, showing that -within 48 hours after right colectomy for cancer- plasmatic concentrations of some proinflammatory cytokines, namely IL-6 and IL-8, even though both increased after operation, were significantly lower in patients receiving laparoscopic resection in comparison with open surgery. Therefore patients subjected to conservative procedures revealed a systemic inflammatory response of lesser degree, as expected [13]. Interestingly -in our study- strongest differences between open conventional and minimally invasive surgery were almost exclusively observed in the early stages of right colorectal cancers, whereas cytokine detection in advanced disease shows a clear postoperative increase, independently of the type of surgical approach. This may be related to a kind of long-term immunostimulation provided by the tumor in the patients with advanced stages of disease, whose proinflammatory cytokine levels remained stably higher in the short term pre- as well as postoperative period, compared to patients with early stage of disease. Hence, present results provide novel evidence that postoperative cytokine production in these patients depends not only on the extent of surgical trauma, but also on tumor stage of disease. Our observations are not always in agreement with previous reports indicating no significant increments in postsurgical cytokine levels amenable to tumor stage of disease [20], [23]. However, an earlier study [46] showed that -besides a general increase of inflammatory markers (CRP, IL-1, IL-6, IL-8 and TNFα) following surgery- specifically for IL-6, postsurgical levels progressively normalized after few days, except for patients with advanced stage of CRC, corroborating our results. In addition, a recent publication [25] demonstrated a minimal immunological advantage in patients -subjected to laparoscopic versus open colectomy- with advanced stage CRC, suggesting that those differences could result more relevant if measured in early stages of CRC. Moreover, we should underline that our analysis was performed exclusively on patients affected by right-sided colorectal cancers. Since several authors reported different outcome of right- versus left-sided colon cancers [4], [5], the involvement of other biological and largely unexplored factors, accounting for this diverse prognosis, is possible.

Contrary to plasmatic levels, no significant differences in cytokine levels were observed in the whole collection of peritoneal fluids, performed during 48 hours following resection. In keeping with previous reports, our data show that -in the early postoperative period- cytokines levels in peritoneal drain fluids were significantly higher than plasma levels, but with no difference between laparoscopic and open colectomy [31], [32]. It could be explained because the magnitude of surgical resection, is similar in both procedures and therefore the cytokine peritoneal amounts may exclusively depend on the local response to the surgery, as previously suggested [31].

In order to measure in vitro the PBMC activity, we stimulated the cells with LPS to examine their cytokine production capacity. Minimally invasive methods seem to minimize postsurgical immunosuppression. In fact, LPS stimulation resulted more efficient in PBMC derived from presurgical blood and minimally invasive surgery than conventional surgery, and PBMC cells derived from patients subjected to laparoscopy produced IL-6 levels significantly higher compared to open surgery. This is in agreement with previous reports [13], [20] performed on human cultured cells, whereas conflicting results were obtained in animal models [47]. Interestingly, in our study, the functional activity of cultured PBMC also seems better preserved in cells derived from early stages of right colorectal cancers, indicating that minimally invasive surgery as well as earlier stages of tumor may correlate with a better preservation of immunological homeostasis (as already proposed [13]). Data on the long-term effects of laparoscopic and open surgery in the specific set of patients undergoing right colectomy for CRC would help to clarify if any difference on tumor–related mortality after the two techniques and between early or advanced stage of disease can be observed, as it is still matter of debate [48]–[51].

## Conclusions

Therefore, if future researches confirm the growing evidence that open methods are associated with a longer compromission of the patient’s immunological defenses, promising perspectives could be considered. The present study suggests that laparoscopic compared to open right resection is a less traumatic procedure with more rapid immunological recovery, mainly in early compared to advanced stages of CRC. Further investigations to better evaluate the long-term oncological advantages of this procedure will be of great interest.

Taken together, these observations corroborate the emerging evidence that a perioperative immunological monitoring may contribute to a more exhaustive prognostic evaluation in CRC.
